# *Zizyphus lotus *L. (Desf.) modulates antioxidant activity and human T-cell proliferation

**DOI:** 10.1186/1472-6882-10-54

**Published:** 2010-09-24

**Authors:** Chahid Benammar, Aziz Hichami, Akadiri Yessoufou, Anne-Marie Simonin, Meriem Belarbi, Hocine Allali, Naim A Khan

**Affiliations:** 1 Laboratoire des Produits Naturels (LAPRONA), Département de Biologie Moléculaire et Cellulaire, Faculté des Sciences, Université Abou Bekr Belkaid, (Tlemcen), Algeria; 2 Laboratoire UPRES-EA4183, Lipides et Signalisation Cellulaire, Université de Bourgogne, (Dijon), France; 3 Laboratoire de Chimie Organique, Substances Naturelles et Analyses (COSNA), Département de Chimie, Faculté des Sciences, Université Abou Bekr Belkaid, (Tlemcen), Algeria

## Abstract

**Background:**

*Zizyphus lotus *L. (Desf.) also known as Jujube, is a deciduous shrub which belongs to Rhamnaceae family. This plant is used in Algerian traditional medicine for its anti-diabetic, sedative, analgesic, anti-inflammatory and hypoglycaemic activities. In the present study, we determined the concentrations of different vitamins (vitamin A, C and E) and fatty acids in root, stem, leaves, fruit pulp and seed of *Zizyphus lotus *L. (Desf.) and assessed the effects of their aqueous extracts on antioxidant status and human T-cell proliferation.

**Methods:**

Aqueous filtrates from different parts, *i.e*, root, leaf, stem, fruit pulp and seed, of *Zizyphus lotus *L. (Desf.) were prepared. Vitamin C levels were determined by precipitating with 10% trichloroacetic acid and vitamin A and E were assessed by HPLC. Lipid composition of these extracts was determined by gas-liquid chromatography. Anti-oxidant capacity was evaluated by using anti-radical resistance kit [Kit Radicaux Libres (KRL^@^; Kirial International SA, Couternon, France)]. T-cell blastogenesis was assessed by the incorporation of ^3^H-thymidine. IL-2 gene expression was evaluated by RT-qPCR.

**Results:**

Our results show that fruit pulp contained higher vitamin A and C contents than other parts of the plant. Furthermore, the fruit pulp was the richest source of linoleic acid (18:2n-6), a precursor of n-6 fatty acids. Fruit seeds possessed higher vitamin C levels than leaves, roots and stem. The leaves were the richest source of vitamin E and linolenic acid (18:3n-3), a precursor of n-3 fatty acids. The antioxidant capacity of the different extracts, measured by KRL^@ ^test, was as follows: pulp < seed<leaf<root < stem. As far as T-cell proliferation is concerned, we observed that the different extracts of *Zizyphus lotus *L. (Desf.) exerted immunosuppressive effects.

**Conclusion:**

Seed extracts exerted the most potent immunosuppressive effects on T cell proliferation and IL-2 mRNA expression. The results of the present study are discussed in the light of their use to modulate the immune-mediated diseases.

## Background

*Zizyphus lotus *L. (Desf.) is abundantly present in the Mediterranean region, throughout Libya to Morocco, Algeria and southern European countries like Spain, Sicily, Greece and Cyprus [1]. In Algeria and Tunisia, it is known as 'Sedra'. The fruit is the edible part of the plant by local population. Several parts of *Zizyphus *have been used by traditional and ancestral medicine, both in North Africa and Middle East, for the treatment of several pathologies including digestive disorders, weakness, liver complaints, obesity, urinary troubles, diabetes, skin infections, fever, diarrhoea and insomnia [2,3]. *Zizyphus lotus *L. (Desf.) is used in Algerian traditional medicine for its anti-diabetic, sedative and hypoglycaemic activities [4,5]. The medicinal properties of this plant depend on the part of the plant concerned (root, leaf stalk and pulp or fruit) and the extract used (ethanolic, butanolic etc.). Fruit has been used for its emollient properties, and a mixture of dried leaves and fruits is applied topically in the treatment of boils. Interestingly, the root bark is known for its antidiabetic activity [6]. Butanol extracts of *Zizyphus spina-christi *leaves which are rich in saponin improved the oral glucose tolerance and potentiated glucose-induced insulin release in type II diabetic rats [7].

Anti-inflammatory, analgesic and anti-ulcerogenic activities of *Zizyphus lotus *L. (Desf.) have been demonstrated in rodents [8,9]. Borgi *et al. *[10] have shown that root barks of *Zizyphus lotus *L. (Desf.), given intraperitoneally, showed a significant and dose-dependent anti-inflammatory and analgesic activity in carrageenan-induced paw edema in the rat. Hence, the presence of flavonoids in the *Zizyphus *extracts was supposed to be responsible for these beneficial effects. Besides, several biologically active molecules, particularly cyclopeptide alkaloids, termed lotusiones [11-13] and dammarane saponins have been isolated from this shrub [14]. In fact, cyclopeptides extracted from *Zizyphus lotus *L. (Desf.) exhibited antibacterial and antifungal properties [15].

Disorders in the immune system may be responsible for the onset of different pathological states. The immunodeficient diseases when the immune system is less active than normal, result in recurring and life-threatening infections. On the other hand, an autoimmune disease results from a hyperactive immune system attacking normal tissues as if they were foreign organisms [16]. Common autoimmune diseases include Hashimoto's thyroditis, rheumatoid arthritis, type I diabetes and lupus erythematosus. Further investigation in this field is expected to play a serious role in promotion of health and treatment of diseases. The T-lymphocytes are the principal mediators of immune-mediated diseases. Hence, a modification of T-cell activation will be a valuable tool to disrupt the disease progression.

As far as the activation of immune system by *Zizyphus *species is concerned, not much is known on the subject. Adhvaryu *et al. *[17] have shown that *Zizyphus *extracts alongwith other plants stimulated neutrophil functions and exerted hepatotoxic and immunomodulatory effects in guinea pigs. Chan *et al. *[18] assessed cell signaling mechanisms in T-cells and provided the evidence that a mixture of herbs containing *Zizyphus *extract induced the expression of mitogen-activated protein kinases (*i.e*. ERK, JNK and p38) in T-cells, indicating that the immunomodulatory effects of *Zizyphus *involve the activation of second messenger cascade.

Since *Zizyphus lotus *L. (Desf.) has been shown to modulate different disorders [2,19-21]. We investigated the role of different crude decoction extracts of this plant on human T-lymphocyte proliferation and expression of IL-2 mRNA. We also studied anti-oxidant properties of this plant since anti-oxidants have been considered as immunomodulators [22] and a modification of the both may be a key factor in etiopathogenesis of several diseases. Hence, we determined the contents of different vitamins (A, C, E) which have been considered as anti-oxidant agents.

## Methods

### Chemicals

The HPLC column (HP ODS Hypersil C18), anti-radical resistance kit (Kit Radicaux Libres (KRL^@^) and tocol were purchased from Lara Spiral, France. RPMI-1640, L-glutamine, penicillin-streptomycin and HEPES were bought from Dutcher, France. DNase was procured from Qiagen, USA. The SuperScript II Reverse Transcriptase, trizol, platinum Taq DNA Polymerase, random primers, and oligonucleotides used as primers in the RT-PCR analysis were purchased from Invitrogen, Life Technologies (Cergy Pontoise, France). Agarose was from Promega (Charbonnière, France). All of the solvents and other chemicals were obtained from Sigma, USA.

### Plant material and preparation of the aqueous extracts of *Zizyphus lotus *(L.)

*Zizyphus lotus *L. (Desf.) was collected from south-western part of Algeria (Ain Ouessara and Maessad (willaya de Djelfa) between September and October 2008. The climate is very arid (annual rain fall: around 324 mm according to Office National de Météorologie) with a drought period from half May to half October (5 months). However, we cannot provide the data on the physico-chemical properties of the soil though the climate conditions indicate that the soil belongs to a semi-drought hit area and *Zizyphus *adapts to such kind of soil in different regions of Africa, Australia and Asia [23]. The plant was recognized by a botanist (Pr Benabadji Nouri, Université Aboubekr Belkaïd, Tlemcen) of the Herbarium Center of the Faculty of Pharmacy (Tlemcen) which contained the voucher specimen (ZLI 1320). Mature whole *Zizyphus lotus *L. (Desf.) plants, collected from between September and October 2008, dried at ambient temperature and stored in a dry place prior to use. A 100 g of either of the following parts, *i.e*, root, leaf, stem, fruit pulp and seed, was suspended in 500 ml distilled water and boiled for 30 min. The decoction obtained was filtered, and the filtrate was frozen at -70°C and, later on, lyophilised and stored at ambient temperature until further use. Lyophilised extract was re-suspended in physiological saline solution (NaCl 0.9%) at 1 mg/ml.

### Determination of vitamin C levels

Vitamin C levels were determined in lyophilised extracts using the method of Roe and Kuether [24] by precipitating with 10% trichloroacetic acid and followed by centrifugation. The supernatant (500 μL) was mixed with 100 μL of DTC reagent (2,4-dinitrophenylhydrazine 3%, thiourea 0.4%, and copper sulfate 0.05%) prepared in 9N sulfuric acid, and incubated at 37°C for 3 h. After the addition of 750 μL of 65% (vol/vol) sulfuric acid, the absorbence was recorded at 520 nm.

### Determination of vitamin A and E levels by HPLC

The α-tocopherol (vitamin E) and retinol (vitamin A) were extracted by hexane (1 ml), three times, from 100 mg of lyophilised extract. The hexane phase was dried up under a stream of nitrogen, resuspended in methanol, and quantified by reverse-phase high-performance liquid chromatography [25]. The stationary phase was constituted of greffed silica (C18 column, HP ODS Hypersil C18; 200 mm × 4.6 mm; maintenance temperature of analytical column, 35°C). The mobile phase was a mixture of methanol/water (98/2, v/v) at a flow rate of 1 ml/min. This method was used to quantify both vitamins A and E in a single chromatographic run in the presence of an internal standard, tocol, which was added to the samples before extraction by hexane. The retention time (RT) of vitamins was determined by the injection of the authentic standards of vitamin A (RT around 5 min), tocol (RT around 8 min), and vitamin E (RT around 15 min). The peaks were detected by an ultraviolet detector set at 292 nm for vitamin E and tocol, and at 325 nm for vitamin A.

### Determination of fatty acid composition

The lipids were extracted as described elsewhere [26] from 1 ml solution of lyophilised extracts (1 mg/ml) in the presence of internal standard (C19:0). The lipid extract was dried under nitrogen and saponified and transmethylated at 80°C for 20 min with BF3/methanol (14%) according to Hichami *et al. *[26]. Fatty acid methyl esters were then extracted in the presence of 2 ml of hexane and separated by gas-liquid chromatography (Packard model 417 gas−liquid chromatograph (Packard, Downers Grove, IL, USA) equipped with flame ionization detector set at 240°C and a 30-m capillary glass column coated with Carbowax 20 M (Applied Science Labs, State College, PA, USA). Helium was used as carrier gas, with a flow rate of 0.4 ml/min. Analysis of fatty acids peaks was achieved with reference to retention time of authentic standards (68b; Nu-Chek-Prep, Elysian, MN, USA) by using DELSI ENICA 31 (Delsi Nermag, Rungis, France). The fatty acid levels were expressed as g per 100 g of lyophilised extract of the plant.

### Antioxidant capacity

The effects of the plant extracts on the sensitivity to free radical aggression was tested by the capacity of red blood cell (RBC) to withstand free radical-induced haemolysis and was measured according to the method of [27], who have clearly demonstrated that, if at least 1 component of the antiradical detoxification system (antioxidants, enzymes) is impaired, a shift of the haemolysis curve is observed toward shorter times. Briefly, washed RBCs were diluted (1:40, vol/vol) with anti-radical resistance [Kit Radicaux Libres (KRL^@^; Kirial International SA, Couternon, France)] buffer (300 mOsmol/kg) and 50 μl of RBCs suspension was assayed in a 96-well microplate coated with a free radical generator (GRL, Kirial International SA). The kinetic of RBCs resistance to hemolysis was determined at 37°C by continuous monitoring of changes in absorbance at 620 nm. The time to reach 50% of total haemolysis was retained for group comparisons.

### Cell culture

The human (Jurkat) T-cells were routinely cultured in RPMI-1640 medium supplemented with 10% foetal calf serum (FCS), 2 mM L-glutamine, 50 μg/ml penicillin-streptomycin and 20 mM HEPES at 37°C in a humidified chamber containing 95% air and 5% CO_2 _[28]. Cell viability was assessed by trypan blue exclusion test. Cell numbers were determined by hemocytometer.

### T-cell blastogenesis

Jurkat T-cells (0.1 × 10^6 ^cells/160 μl) were suspended in RPMI-1940 without serum and seeded in 96-well plate (Nunc, Roskilde, Denmark), then cells were incubated for 4 h with increasing concentration of *Zizyphus *(5 μg/ml, 10 μg/ml and 20 μg/ml), then stimulated with anti-CD3 antibodies (30 μg/ml). Cells were distributed in six replicates as follows: 160 μl of cell suspension, 20 μl of *Zizyphus *extract and 20 μl of anti-CD3 antibodies as described elsewhere [29]. After 36 h, 20 μl of [^3^H] thymidine (20 Ci/mmol, 0.5 μCi/well) was added and, 12 h later, the cells were harvested with a cell harvester (Dynatech, Burlington, MA, USA), trapping their DNA onto glass filtermats. Dried filter circles were placed in plastic minivials (Pakard, Downers Grove, IL, USA), 2 ml of Optifluor-O (Pakard) was added, and the radioactivity was recorded in a scintillation counter (Beckman, Fullerton, CA, USA).

### RNA isolation and real time quantitative PCR

Cells were cultured as described above in the presence of *Zizyphus *extracts and stimulated with anti-CD3 antibodies for 2 h [30]. Total RNA from cells was extracted using trizol and underwent DNase treatment using the RNase-free DNase Set (Qiagen). One μg of total RNA was reverse transcribed with Super script II H-reverse transcriptase using oligo (dT) according to the manufacturer's instructions. Real time PCR was carried out on the iCycler iQ real time detection system and amplification was undertaken by using SYBR Green I detection. Oligonucleotide primers were as follow: beta-actin forward: 5'-ATGATATCGCCGCGCTCGTCGTC-3', beta-actin reverse 5'-AGGTCCCGGCCAGCCAGGTCCAG-3'; IL-2 forward 5'-CACTAATTCTTGCACTTGTCAC-3', IL-2 reverse 5'-CCTTCTTGGGCATGTAAAACT-3'. IL-2. The Amplification was carried out in a total volume of 25 μl containing 12.5 μl SYBR^® ^Green supermix, *i.e.*, PCR buffer [50 mM KCl, 20 mM, Tris-HCl (pH 8.4), 3 mM MgCl_2_], 0.2 mM each dNTPs, 0.63 U iTaq DNA polymerase, SYBR green 1,10 nM fluoresein, and 12.5 μl containing 0.3 μM each primer and diluted cDNA.

The conditions of amplification consisted of an initial denaturation step at 95°C for 5 min as a "hot start" followed by 40 cycles at 95°C for 30 s/60°C for 30 s with a single fluorescence detection point at the end of the relevant annealing or extension segment. At the end of the PCR, the temperature was increased from 60 to 95°C at a rate of 2°C/min, and the fluorescence was measured every 15 s to construct the melting curve. The standard curves were generated for each gene using serial dilutions of positive control template in order to establish PCR efficiencies. All determinations were performed, at least, in duplicates using two dilutions of each assay to achieve reproducibility.

Results were evaluated by iCycler iQ software including standard curves, amplification efficiency (E) and cycle threshold (Ct). Relative quantification of mRNA in different groups was determined as follows: ΔΔCt = ΔCt of gene of interest - ΔCt of beta actin. ΔCt = Ct of treated cells - Ct control cells. Relative quantity (RQ) was calculated as follows: RQ = (1+E)^(-ΔCt) ^.

### Statistical analysis

Statistical analysis of data was carried out using Statistica (version 4.1, Statsoft, Paris, France). The significance of the differences between mean values was determined by analysis of variance one way, followed by a least-significant-difference (LSD) test. For all the tests, the significance level chosen was p < 0.05. The Spearman's rank test was employed for the correlation coefficients.

## Results

### Concentrations of vitamin A, C and E in different parts of *Zizyphus lotus *L. (Desf.)

Table 1 shows that concentration of vitamin A and C was higher in fruit pulp than those of the leaves, root and stem of the *Zizyphus lotus *L. (Desf.). Interestingly, vitamin A could not be detected in fruit seeds, though they contained significant amounts of vitamin C. The concentration of vitamin A and C in other parts of the plant were as follows: pulp < leaves<root < stem while we compared these four parts of the plant. *Zizyphus *leaves contained higher vitamin E concentrations than root, fruit pulp, stem and fruit seed. The root and stem contained the similar levels of vitamin E. The vitamin E concentrations in fruit pulp and seed were not statistically different (Table 1).

**Table 1:** Contents of three vitamins in the different parts of *Zizyphus lotus *(L.)

Vitamins	Leaves	Root	Pulp	Stem	Seed
(mg/100 g of dry weight)					
Vitamin A	13.52 ± 0.06^(a)^	6.45 ± 0.09^(b)^	71.63 ± 1.23^(c)^	3.8 ± 0.96^(d)^	nd
Vitamin C	63.40 ± 1.23^(e)^	47.20 ± 0.82^(f)^	190.65 ± 1.48^(g)^	24.65 ± 1.22^(h)^	170.84 ± 0.63^(i)^
Vitamin E	155.71 ± 1.08^(j)^	4.7 ± 0.23^(k)^	11.23 ± 1.36^(l)^	4.5 ± 0.097^(m)^	9.2 ± 0.54^(n)^

Vitamin concentrations were determined as described in the materials and methods section. Values are means ± SEM. Each value represents the mean of three determinations. Significant difference between different groups is as follows: a vs b p < 0.01; d vs a, b p < 0.01; c vs a, b, d p < 0.001; e vs f p < 0.01; g vs e, f, h, I p < 0.001; j vs k, l, m, n p < 0.001. k vs m, not significantly different; nd = not detected

### Fatty acid composition of different parts of *Zizyphus lotus *L. (Desf.)

A perusal of Table 2 shows that fruit seeds were richer in fatty acids than other parts of the plant. Besides, the *Zizyphus *plant seems to be a good source of saturated (16:0 and 18:0), monounsaturated (18:1n-9) and polyunsaturated (18:2n-6) fatty acids. As far as the essential fatty acids are concerned, linoleic acid was present in all parts of the plant, where the fruit pulp was found to be the richest source. Linolenic acid could not be detected in stem, fruit pulp and root of the *Zizyphus *(Table 2). Eicosatrienoic acid (20:3n-3), an intermediate agent between linolenic acid and docosahexaenoic acid, was present only in fruit seed, stem and root. Arachidonic acid was detected only in leaves of the *Zizyphus*. Other fatty acids were present principally in fruit seed and leaves (Table 2).

**Table 2:** Fatty acid composition (g/100 g) of different parts of *Zizyphus lotus *(L.)

Fatty acids	Seeds	Leaves	Stem	Pulp	Root	Statistiques
14:0	0.15 ± 0.028	0	0	0	0	
16:0	10.8 ± 1.80^a^	43.41 ± 1.82^b^	33.80 ± 1.95^c^	27.59 ± 1.77^d^	38.76 ± 1.59^e^	b:a, c, d, e p < 0.001
16:1	0.130 ± 0.22^a^	5.96 ± 0.63^b^	0	0	0	a:b p < 0.001
18:0	5.45 ± 1.50^a^	22.15 ± 1.31^b^	24.40 ± 1.62^c^	11.25 ± 1.31^d^	22.00 ± 0.69^e^	c:d, a p < 0.001
18:1n-9	62.79 ± 1.33^a^	6.30 ± 1.50 ^b^	21.73 ± 194^c^	24.52 ± 0.13^d^	19.73 ± 1.80^e^	a:b, c, d, e p < 0.001
18:2n-6	14.22 ± 1.96 ^a^	6.20 ± 1.67^b^	11.10 ± 1.72^c^	36.63 ± 1.26^d^	13.24 ± 0.11^e^	d:a, b, c, e p < 0.001
18:3n-3	1.30 ± 0.64^a^	9.15 ± 1.95^b^	0	0	0	a:b p < 0.001
19:0	0	0	0	0	0	
20:1	3.12 ± 1.40^a^	2.17 ± 0.31^b^	0	0	0	a:b NS
20:2n-9	0.20 ± 0.057^a^	1.53 ± 0.60^b^	0	0	0	a:b NS
20:3n-3	0.83 ± 0.035^a^	0	8.95 ± 0.91^b^	0	2.59 ± 0.63^c^	b: a, c p < 0.001
20:4n-6	0	1.58 ± 0.95	0	0	0	
20:0	0.1 ± 0.058	0	0	0	0	
24:0	0.9 ± 0.92^a^	1.54 ± 0.61^b^	0	0	3.66 ± 1.18^c^	c:a, b p < 0.001

Different fatty acids were determined as described in materials and methods section. Values are means ± SEM. Each value represents the mean of three determinations. NS = insignificant differences

### Antioxidant capacity

**Figure 1:**
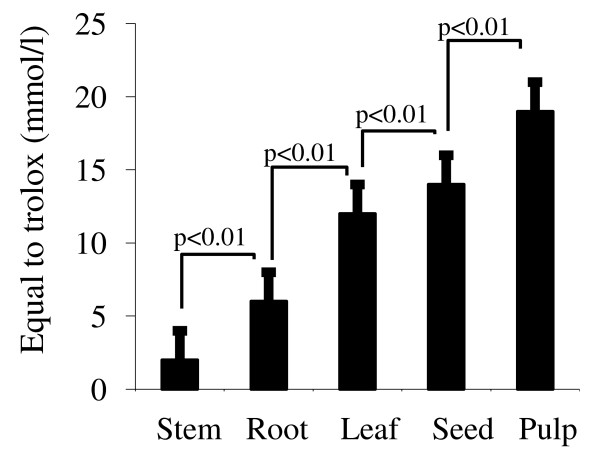
**Different extracts of the *Zizyphus *exhibit antioxidant capacity**. Results are presented as mean ± S.D. Each value represents the mean of six determinations.

If the fruit pulp exhibited higher antioxidant capacity than other parts of the plant, then the order of antioxidant capacity should be pulp < fruit seed<leaves<root < stem (Figure 1).

### Effects of *Zizyphus *on T-cell blastogenesis and IL-2 expression

To assess the toxicity of the *Zizyphus lotus *L. (Desf.), we used the trypan blue exclusion test. We observed that *Zizyphus lotus *L. (Desf.) extracts were cytotoxic only beyond the concentration of 40 μg/ml. Since there was no cytotoxic effect from 10 μg/ml to 30 μg/ml of the plant extract and there was no significant difference between 20 μg/ml and 30 μg/ml, we show the effects 5 μg/ml, 10 μg/ml and 20 μg/ml of the extracts in our study.

**Figure 2:**
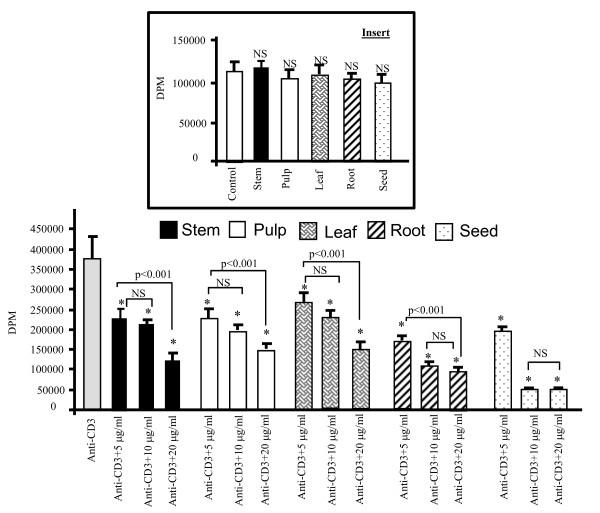
**Different extracts of the *Zizyphus *modulate T-cell proliferation**. Results are presented as mean ± S.D. The control cells without anti-CD3 antibodies had only 2000 ±120 DPM. Values differ significantly compared with anti-CD3-stimulated cells p < 0.001 (*). Each value represents the mean of six determinations. The insert shows that effects of different extracts at 20 μg/ml in the absence of anti-CD3 antibodies.

The anti-CD3 antibodies activated human T-cell line in a highly significant manner. All the extracts significantly diminished the T-cell proliferation, activated by exogenous anti-CD3 antibodies (Figure 2). Though all the concentrations of the extracts inhibited T-cell activation, the highest inhibitory effect was noticed at 20 μg/ml. In fact, there was no significant difference of the response between 20 μg/ml and 30 μg/ml (not shown). Figure 2 insert shows the effect at 20 μg/ml in T-cells without stimulation.

**Figure 3:**
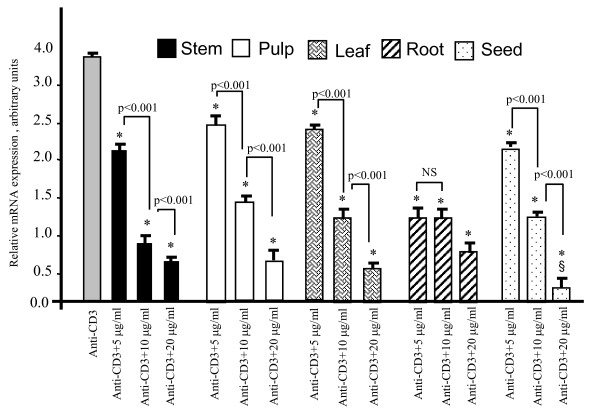
**Different extracts of the *Zizyphus *modulate IL-2 mRNA expression**. The human Jurkat T-cells (1 × 10^6^/assay) were incubated in the presence of various extracts of the *Zizyphus lotus *L. (Desf.) plant and stimulated with anti-CD3 antibodies during 2 h. Values differ significantly compared with anti-CD3-stimulated cells p < 0.001 (*). Each value represents the mean of six determinations. § represent the significant differences (p < 0.01) with stem, pulp, leaf, root extract at 20 μg/ml.

We also assessed the expression of IL-2 mRNA and we noticed that different extracts of the *Zizyphus *exerted inhibitory effects (Figure 3). The most potent inhibitory effect was observed at 20 μg/ml and there was no significant difference between this dose and 30 μg/ml (not shown).

## Discussion

T-cell abnormalities are believed to be the major cause of autoimmune diseases like type 1 diabetes. In type 2 diabetes also, the inflammation leading to the activation of monocytes is postulated to be important for enhancing insulin resistance and contributing to the loss of insulin secretary function by islet cells [16].

Jujube has increasingly become popular as a source of food and medicine for thousands of years [19,31]. The beneficial effects may be related to the presence of biologically active compounds [32]. In our study, we have observed that different extracts of the *Zizyphus *exerted the antioxidant activity. Hence, it is possible that the antioxidant activity of *Zizyphus lotus *L. (Desf.) might be due to the presence of different vitamins. The *Spearman's correlation *coefficient (Rs) between antioxidant activity and vitamins or fatty acids are as follows: vitamin A vs antioxidant Rs = 0.95; vitamin C vs antioxidant Rs = 0.82; vitamin E vs antioxidant = Rs 68; n-fatty acids vs antioxidant Rs = 0.27. Furthermore, Lenucci et al. [33] have demonstrated that antioxidant activity is likely due to the presence of ascorbic acid, tocopherol and pigments. This argument also supports the highest antioxidant activity of the pulp which contains the highest contents of vitamin A and C and a substantial quantity, though lesser than leaves, of vitamin E. The seed extract, which contains higher vitamin C than stem, root leaves, stands at the second place as far as the antioxidant activity is concerned. The leaf extract which contains vitamin E in the highest quantity, but lesser vitamin C and A than pulp, stands at the third place regarding the antioxidant activity. It is also possible that the antioxidant activity, in part, of the pulp might be due to the presence of polyphenols as suggested by Lamia-Meda *et al. *[34] who have recently reported that *Zizyphus mauritania *(L.) is rich in these agents. These investigators further assessed the antioxidant capacity of these extracts and finally concluded that the fruit, rich in polyphenols, was responsible for the antioxidant property. However, we did not determine the concentrations of polyphenols in our extracts. Nonetheless, our observations suggest that *Zizyphus lotus *L. (Desf.) is a good source of antioxidant agents.

The present study also shows that *Zizyphus *decoction exerted an immmunosupressive activity. Our results agree well with the findings of Adhvaryu *et al. *[17] who have also observed that *Zizyphus *extracts exert immunomodulatory effects in guinea pigs. The induction of IL-2 gene transcription is a critical event for T-cell proliferation and effector functions. We observed that different extracts inhibited T-cell blastogenesis and IL-2 mRNA expression. However, the seed extract was found to be the most potent immunosuppressor as this extract, at 10 μg/ml concentrations, inhibited by 86 ± 1.2% of T-cell proliferation whereas the extracts of stem, pulp, leaf and root inhibited, respectively, the same by 38 ± 4.2%, 43 ± 4.4%, 29 ± 5.2% and 72 ± 4.1%. The significant immunosuppressive effect of seed extract cannot be attributed to the presence of vitamins as this extract did not contain vitamin A, and vitamin E concentration was lesser than that in pulp and leaf, and vitamin C was lesser than that in the pulp fractions.

Hence, it is possible that the fatty acids might be responsible for this immunosuppressive effect. The *Spearman's correlation *coefficient between T-cells proliferation (TCP) and vitamins or fatty acids are as follows: vitamin A vs TCP Rs = 0.0037; vitamin C vs TCP Rs = 0.19; vitamine E vs TCP Rs = 0.15; n-3 fatty acids vs TCP Rs = 0.70. Indeed, seed fraction was the richest in fatty acids and it contained, notably, three immunosuppressive n-3 fatty acids (18:3 n-3, 20:3 n-3 and 20:03). Silva *et al. *[35] have reported that the oil of *Zizyphus mistol *was rich in n-3 fatty acids (18:3 n-3) and, therefore, modulated tumor growth in animal models [36]. Zaho *et al. *[37] identified eleven components in a product of *Zuzyphus jujuba*, cultivated in China, but they failed to detect linolenic acid (18:3 n-3). However, Guil-Guerrero *et al. *[38] quantified all of the fatty acids which we report in the present study, in *Zuzyphus jujuba *cultivated in Spain. In fact, the fatty acid composition depends on fruit variety, culture type (irrigation or not), location, and developmental stage (mature or raisin) which may vary from one country to another.

It has been well established that n-3 fatty acids exert immunosuppressive and anti-inflmmatory activities both in experimental and clinical studies [39]. Indeed, the extracts of *Zizyphus lotus *L. (Desf.) have been shown to possess anti-inflammatory properties [19]. It is noteworthy that the *Zizyphus *extracts in the absence of anti-CD3 antibodies failed to inhibit cell proliferation, suggesting that *Zizyphus *extracts under normal conditions do not modulate T-cell proliferation. These observations are in analogy to the n-3 fatty acids which, being authentic immunosuppressors, failed to influence normal T-cell proliferation in healthy subjects [40]. In fact, the best immunosuppressors might interfere, principally, with abnormal T-cell activation, as seen in the autoimmune diseases, without influencing the same in healthy situations [41]. As far as the mechanism of action of fatty acids is concerned, it has been well established that they interfere with cell signalling, particularly with the cascade of MAP kinases like ERK1/2 and p38 [42,43]. In fact, Chan *et al. *[18] have also demonstrated that a mixture of herbs containing *Zizyphus *extracts also interfere with the phosphorylation of ERK1/2 and p38 in T-cells.

## Conclusions

To sum up, we can state that different aqueous extracts of *Zizyphus lotus *L. (Desf.) bear therapeutic potential as they possess antioxidant and immunosuppressive properties. To our knowledge, no study has, as yet, been carried out on the effects of *Zizyphus lotus *L. (Desf.) in autoimmune diseases. However, a mixture of plants of Chinese medicine containing *Zizyphus jujube *L. (Desf.) has been found to modulate immune system [18]. Further studies are required to elucidate the effects of different extract of this plant in the progression of autoimmune diseases or organ transplantation.

## Competing interests

The authors declare that they have no competing interests.

## Authors' contributions

CB, AH and NAK designed the study. AY and AMS participated in the technical work. MB and HA supervised the plant collection. NAK wrote the MS. NAK and AH established the collaborative work with Algerian team. All authors have read and approved the final manuscript.

## Pre-publication history

The pre-publication history for this paper can be accessed here:

http://www.biomedcentral.com/1472-6882/10/54/prepub
